# The Accumulation of Key Stroke Risk Factors and Its Association With the Characteristics of Subjects: A Population Based Cross Sectional Study

**DOI:** 10.3389/fneur.2018.00949

**Published:** 2018-11-12

**Authors:** Peng Zhang, Hang Jin, Zhen-Ni Guo, Hui-Jie Sun, Fu-Liang Zhang, Xin Sun, Yi Yang

**Affiliations:** ^1^Clinical Trial and Research Center for Stroke, Department of Neurology, The First Hospital of Jilin University, Changchun, China; ^2^Department of Neurology, The First Hospital of Jilin University, Changchun, China; ^3^Cadre Ward, The First Hospital of Jilin University, Changchun, China

**Keywords:** stroke, risk factors, life style, accumulation, prevention

## Abstract

**Background:** Evidence has shown that the greater the accumulation of risk factors for stroke, the greater the risk of stroke. Early intervention in the accumulation of risk factors for stroke can effectively reduce the incidence of stroke. The study aimed to investigate the distribution of the number of certain risk factors for stroke (hypertension, hyperlipidemia, overweight and obesity, and diabetes) and to explore the cause of the accumulation of certain stroke risk factors.

**Methods:** A total of 4,052 participants aged 40 years or older were selected by the multistage stratified cluster sampling method in Dehui City in Jilin province, China. Descriptive data analyses were conducted. Multiple regression analyses were used to explore the adjusted association between the accumulation of key stroke risk factors and subjects' lifestyle and demographic characteristics.

**Results:** Overall, 84.1% of the participants in this study had one or more of the four certain risk factors for stroke. The odds ratios (ORs) and 95% confidence intervals (CIs) of having ≥1, ≥2, and ≥3 key stroke risk factors were 1.627 (1.258, 2.103), 1.446 (1.209, 1.728), and 1.394 (1.164, 1.670), respectively, for males compared to females. Similarly, the ORs and 95% CIs of having ≥1, ≥2, and ≥3 key stroke risk factors were 1.227 (1.009, 1.492), 1.256 (1.096, 1.442), and 1.450 (1.262, 1.667), respectively, for partially salty diets compared to normal diets. Compared to people who did not exercise regularly, the ORs and 95% CIs of having ≥1, ≥2, and ≥3 key stroke risk factors were 0.693 (0.544, 0.883), 0.800 (0.679, 0.944), and 0.775 (0.659, 0.913), respectively, for people who regularly exercised. Compared to people who without a family history of cerebrovascular diseases, the ORs and 95% CIs were 1.418 (1.162, 1.732), 1.327 (1.154, 1.525), and 1.209 (1.050, 1.393), for people who with it.

**Conclusions:** Male, partially salty diets, and family history of cerebrovascular diseases were risk factors for the accumulation of certain stroke risk factors while regular physical exercise was a protective factor.

## Introduction

Stroke is typically characterized as a neurological deficit attributed to an acute focal injury of the central nervous system by a vascular cause (1), and is the main cause of long-term adult disability and the second major cause of death in China (2, 3). Although patients received standardized stroke treatment, many suffered from residual disabilities or cognitive deficits. Therefore, early prevention is essential to reducing the burden of stroke.

Blood pressure, blood lipids, blood glucose, and BMI are indicators that can be objectively measured in cross-sectional studies. Therefore, recall bias and other possible information bias can be effectively avoided. Hypertension, overweight or obesity, diabetes, and hyperlipidemia are well-established major risk factors for stroke with high population attributable risk (3). At the same time, the prevalence of these four diseases is high in Jilin province (4). Therefore, hypertension, overweight or obesity, diabetes, and hyperlipidemia are considered to be the key risk factors for stroke in this study. Evidence has shown that the greater the accumulation of risk factors for stroke, the greater the risk of stroke (5). Accordingly, early intervention in the accumulation of risk factors for stroke can effectively reduce the incidence of stroke. At present, studies regarding the influence of demographic characteristics and lifestyle on the aggregation of stroke risk factors are relatively rare.

This population-based cross-sectional study was part of the Stroke Screening and Prevention Programme of the National Health and Family Planning Commission of China. The survey was conducted in 2016 and supervised by the National Center for Stroke Control and Prevention. In this study, we explored the distribution of the number of risk factors for stroke. Adjusted associations between the number of accumulated stroke risk factors and demographic characteristics and lifestyle were also examined in order to clarify the strength of association.

## Materials and methods

### Study design and population

This population-based cross-sectional study was conducted among residents aged 40 years or older and were living in Dehui City for over 6 months in 2016. The multistage stratified cluster sampling method was used to select the study sample in Dehui City in Jilin province, China. In the first stage, the districts of Dehui city were divided into rural and urban area. In the second stage, 30 villages (rural) and 10 towns (urban) were randomly selected using probability proportional to size (PPS) sampling. In the third stage, 4 or 5 communities were sampled from both urban and rural area using PPS. Finally, 1 adult resident was randomly selected from each household of the selected communities (6). A total of 4,445 subjects were recruited and 4,100 completed the survey, a response rate of 92.23%. After excluding the subjects without complete information of values, 4,052 subjects were included in this study.

### Ethical standards

This study was approved by the human ethics and research ethics committee of the First Hospital of Jilin University (approval No: 2015-R-250), and written informed consent was obtained from all the participants.

### Data collection

All data were collected by an interviewer administered questionnaire. The questionnaire was composed of three parts: general information (socio-demographic characteristics and health-related information), body measurements (such as height, weight, and blood pressure), and laboratory results (such as blood lipids). The investigators had been uniformly trained and followed the same questionnaire instruction. Eligible investigators had to pass an examination at the end of training.

### Measurements

The subjects' heights and weights were measured without shoes and wearing light clothing according to a standardized protocol and technique. The accuracy of the measurement were 0.1 cm and 0.1 kg, respectively. Blood pressure was measured using an electronic sphygmomanometer (OMRON HEM-7200). Each participant rested for at least 20 min before measurements were taken, and the average value of two measurements was adopted. The blood samples were obtained in the morning from subjects after fasting for at least 8 h and transported to a clinical laboratory (Changchun Kingmed Centre for Clinical Laboratory Co. Ltd.). The blood samples were examined within 8 h to measure fasting blood glucose (FBG), total cholesterol (TC), triglyceride (TG), low-density lipoprotein cholesterol (LDL-C), and high-density lipoprotein cholesterol (HDL-C).

### Definitions

The diagnosis of hyperlipidemia was based on the criteria of the “Chinese Guidelines on Prevention and Treatment of Dyslipidemia in Adults”: TC ≥ 5.18 mmol/L or TG ≥ 1.70 mmol/L or HDL-C < 1.04 mmol/L or LDL-C ≥ 3.37 mmol/L or previous diagnosis of hyperlipidemia by a physician (7). Those previously diagnosed by a physician or those with a fasting plasma glucose (FPG) of ≥ 7.0 mmol/L were defined as diabetic (8). Systolic ≥ 140 mmHg or diastolic ≥ 90 mmHg and/or self-reported hypertension were regarded as hypertension (9). According to the criteria of weight for Chinese adults, 24 ≤ BMI < 28 were defined as overweight, and 28 ≤ BMI were defined as obesity (10). Smoking was defined as having smoked one or more cigarettes every day for more than 6 months. Those who had never smoked were defined as non-smokers. Those who have been smoking for < 6 months were also defined as non-smokers. Those who had never smoked but having been passively exposed to tobacco smoke were defined as passive smokers (11). Drinking was defined as consuming more than 3 alcoholic drinks per day or 7 drinks per week according to the National Institute on Alcohol Abuse and Alcoholism (NIAAA) guidelines (that is, any drink containing 14 g of pure alcohol) (12). Irregular exercise was defined as physical exercise < 3 times a week for < 30 min each session, and this included industrial and agricultural labor. A balance diet and salty diet were defined according to “Dietary guidelines for Chinese Residents (2007)” (13). Direct relatives in the three generation suffered from cerebrovascular diseases (Cerebral atherosclerosis, thrombosis, and stroke etc.) were defined as having a family history of cerebrovascular disease.

### Statistical analysis

Descriptive data analyses were conducted. Frequency distribution was used to present the subjects' characteristics, and the prevalence of stroke risk factors were reported using percentages. The chi-squared test was used to compare the prevalence of stroke risk factors in different age groups. The result of the trend test was analyzed using linear-by-linear association. Cramer's V or Gamma was used to indicate the relationship between the participants' characteristics and the number of key stroke risk factors. Multiple regression analyses were used to explore the adjusted association between the participants' characteristics and the number of key stroke risk factors. All tests were two-tailed, and *P* < 0.05 was considered statistically significant. Data analyses were conducted using SPSS 22.0 (IBM Corp., Armonk, NY, USA).

## Results

A total of 4,052 participants aged 40 years or older were eventually included in this study. The mean age of the participants was 54.85 ± 9.30, 40.0% were male, and 51.0% were urban residents. Supplementary Table I showed that the majority were between 40 and 49 years old and had a junior middle-school education. The smoking rate was 33.9% and the drinking rate was 26.5%. Overall, 77.7% of the participants exercised regularly, 38.7% preferred salty diets, and 34.5% had family history of cerebrovascular diseases. Most of the participants had a balanced diet (58.2%) and ate fruit ≥5 times per week (88.3%).

**Figure 1:**
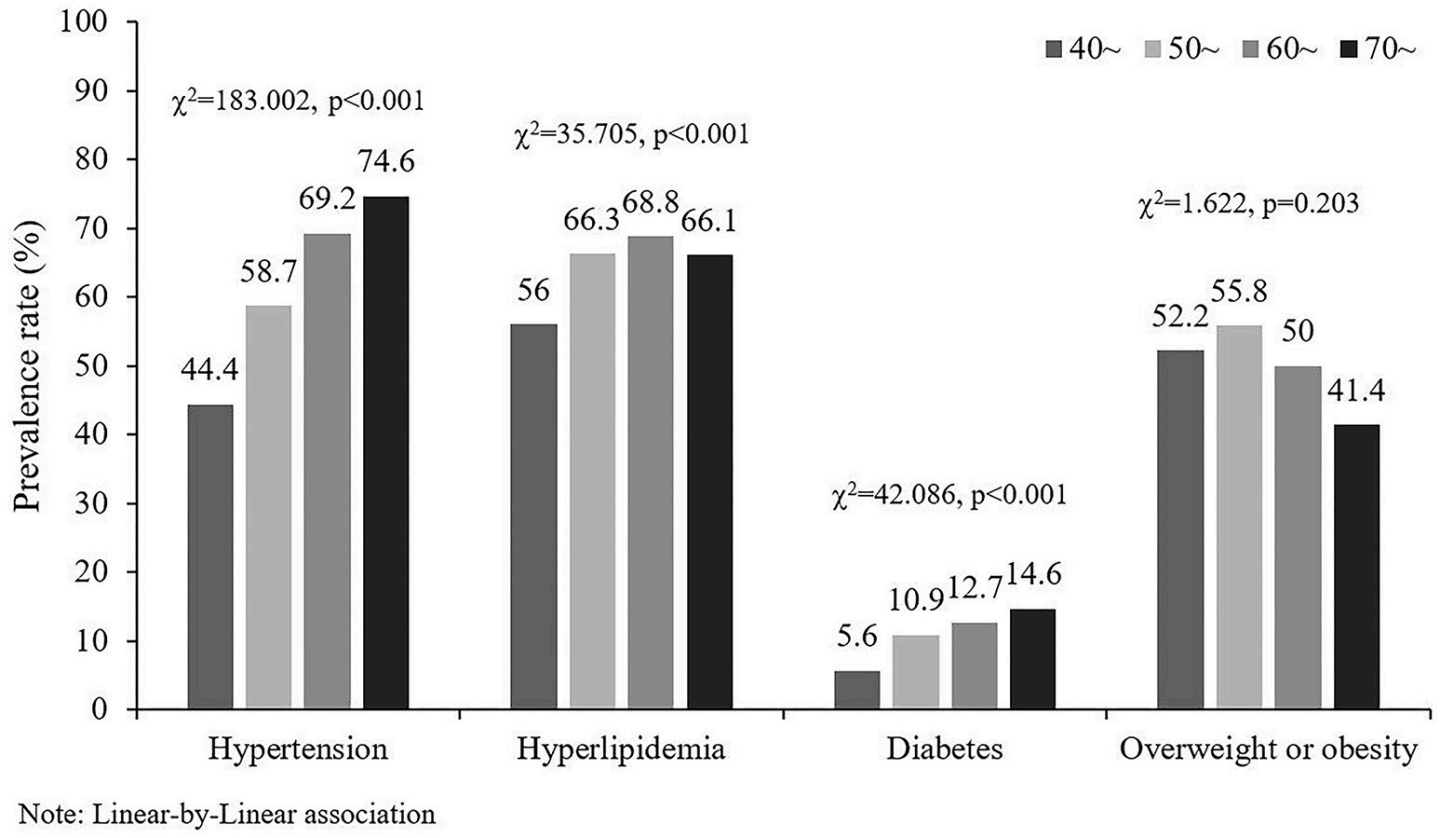
The prevalence rate of stroke risk factors in different age groups.

The prevalence of hypertension and diabetes were the highest in the 70~ year-old group, while hyperlipidemia was highest in the 60- to 69-year-old group (Table 1). The lowest prevalence of hypertension, hyperlipidemia, and diabetes were in 40- to 49-year-old group. Figure 1 showed that the prevalence of hypertension, hyperlipidemia and diabetes had a tendency to increase with age. The chi-squared trends were 183.002, 35.705, and 42.086, respectively, and the *p*-values were all < 0.001.

**Table 1:** The prevalence rate of stroke risk factors in different age groups.

**Risk factors**	**40~**	**50~**	**60~**	**70~**	**Total**	**F/χ^2^**	***p***
**Hypertension**						189.065	< 0.001
Yes	611(44.4)	806(58.7)	698(69.2)	220(74.6)	2335(57.6)	
No	765(55.6)	566(41.3)	311(30.8)	75(25.4)	1717(42.4)	
**Hyperlipidemia**						50.788	< 0.001
Yes	771(56.0)	910(66.3)	694(68.8)	195(66.1)	2570(63.4)	
No	605(44.0)	462(33.7)	315(31.2)	100(33.9)	1482(36.6)	
**Diabetes**						46.527	< 0.001
Yes	77(5.6)	150(10.9)	128(12.7)	43(14.6)	398(9.8)	
No	1299(94.4)	1222(89.1)	881(87.3)	252(85.4)	3654(90.2)	
**Overweight or obesity**						22.110	< 0.001
Yes	718(52.2)	765(55.8)	555(55.0)	122(41.4)	2160(53.3)	
No	658(47.8)	607(44.2)	454(45.0)	173(58.6)	1892(46.7)	
**BMI**	24.47 ±.3.35	24.61 ± 3.32	24.44 ± 3.35	23.53 ± 3.30	24.47 ± 3.35	8.844	< 0.001
**Waist circumference**	83.27 ± 9.33	86.03 ± 8.89	87.97 ± 9.11	86.31 ± 8.61	85.60 5.9.25	54.733	< 0.001
**Regular exercise**						68.424	< 0.001
Yes	1072(77.9)	1133(17.4)	765(75.8)	180(61.0)	3150(77.7)	
No	304(22.1)	239(82.6)	244(24.2)	115(39.0)	902(22.3)	
**Smoking**						37.792	< 0.001
Yes	384(27.9)	508(37.0)	381(37.8)	100(33.9)	1373(33.9)	
No	754(54.8)	641(46.7)	474(47.0)	156(52.9)	2025(50.0)	
Passive	238(17.3)	223(16.3)	154(15.2)	39(13.2)	654(16.1)	
**Drinking**						16.229	0.001
Yes	403(29.3)	370(27.0)	244(24.2)	57(19.3)	1094(26.5)	
No	973(70.7)	1002(73.0)	765(75.8)	238(80.7)	2978(73.5)	
**Family history of cerebrovascular diseases**						13.590	0.004
Yes	504(36.6)	474(34.5)	344(34.1)	75(25.4)	2655(65.5)	
No	872(63.4)	898(65.5)	665(65.9)	220(74.6)	1397(34.5)	
**Family history of coronary artery disease**						14.672	0.002
Yes	379(27.5)	352(25.7)	223(22.1)	57(19.3)	1011(25.0)	
No	997(72.5)	1020(74.3)	786(77.9)	238(80.7)	3041(75.0)	

Table 2 describes the relationship between the participants' characteristics and the number of key stroke risk factors. 24.1% of the participants in this study had one of the four risk factors for stroke; 29.9% had two of the four risk factors for stroke; 27.0% had three of the four risk factors for stroke; 5.1% had four risk factors for stroke. Overall, 84.1% of the participants in this study had one or more of the four certain risk factors for stroke. It also showed that the number of key stroke risk factors for the participants were related to sex, area, age, education, drinking alcohol, partially salty diet, regular exercise, and family history of cerebrovascular diseases. The number of key stroke risk factors were increased with age and decreased with education.

**Table 2:** The relationship between the participants' characteristics and the number of certain stroke risk factors, *n* (%).

**Characteristics**	**0**	**1**	**2**	**3**	**4**	**Cramer's V or Gamma**	***p***
**Total**	565(13.9)	975(24.1)	1211(29.9)	1095(27.0)	206(5.1)	
**Sex**						0.094	< 0.001
Male	175(10.8)	367(22.7)	495(30.6)	499(30.8)	83(5.1)	
Female	390(16.0)	608(25.0)	716(29.4)	596(24.5)	123(5.1)	
**Area**						0.052	0.028
Urban	304(14.7)	455(22.0)	634(30.7)	572(27.7)	102(4.9)	
Rural	261(13.1)	520(26.2)	577(29.1)	523(26.4)	104(5.2)	
**Age (year)**						0.185	< 0.001
40~	302(21.9)	362(26.3)	352(25.6)	323(23.5)	37(2.7)	
50~	147(10.7)	342(24.9)	424(30.9)	383(27.9)	76(5.6)	
60~	94(9.3)	200(19.8)	326(32.3)	313(31.1)	76(7.5)	
70~	22(7.5)	71(24.1)	109(36.9)	76(25.7)	17(5.8)	
**Education**						−0.065	< 0.001
Primary school and below	158(10.9)	363(25.1)	436(30.2)	409(28.3)	80(5.5)	
Junior middle school	252(14.9)	390(23.0)	514(30.3)	452(26.6)	88(5.2)	
Senior middle school	91(16.9)	121(22.5)	156(29.1)	142(26.4)	27(5.1)	
College and above	64(17.2)	101(27.1)	105(28.2)	92(24.6)	11(2.9)	
**Smoking**						0.026	0.701
Yes	183(13.3)	350(25.5)	399(29.1)	368(26.8)	73(5.3)	
No	299(14.8)	471(23.3)	610(30.1)	549(27.1)	96(4.7)	
Passive	83(12.7)	154(23.5)	202(30.9)	178(27.2)	37(5.6)	
**Drinking**						0.071	< 0.001
Yes	118(11.0)	236(22.0)	335(31.2)	331(30.8)	54(5.0)	
No	447(15.0)	739(24.8)	876(29.4)	764(25.7)	152(5.1)	
**Partially salty diet**						0.087	< 0.001
Yes	191(12.2)	359(22.9)	439(28.0)	497(31.7)	81(5.2)	
No	374(15.1)	616(24.7)	772(31.1)	598(24.1)	125(5.0)	
**Regular exercise**						0.066	0.001
Yes	465(14.8)	772(24.5)	939(29.8)	830(26.3)	144(4.6)	
No	100(11.1)	203(22.5)	272(30.1)	265(29.4)	62(6.9)	
**Family history of cerebrovascular diseases**						0.057	0.010
Yes	166(11.9)	315(22.5)	435(31.2)	403(28.8)	78(5.6)	
No	399(15.0)	660(24.9)	776(29.2)	692(26.1)	128(4.8)	
**Dietary pattern**						0.039	0.147
Balanced	336(14.2)	563(23.9)	692(29.3)	657(27.9)	111(4.7)	
More meats	31(11.7)	52(19.7)	80(30.3)	84(31.9)	17(6.4)	
More vegetables	198(13.9)	360(25.2)	439(30.6)	354(24.8)	78(5.5)	
**Fruit consumption (times per week)**						−0.025	0.481
≤ 2	15(13.3)	28(24.8)	31(27.4)	35(31.0)	4(3.5)	
3-4	49(13.6)	77(21.4)	114(31.9)	105(29.2)	14(3.9)	
≥5	501(14.0)	870(24.3)	1066(29.7)	955(26.7)	188(5.3)	

**Figure 2:**
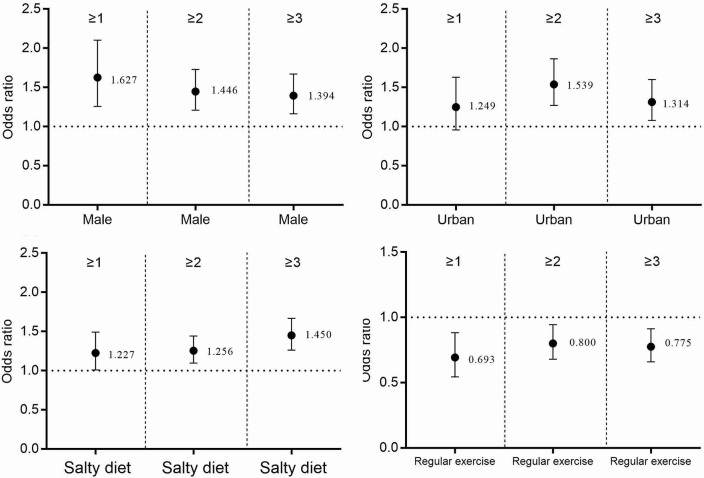
The accumulation of stroke risk factors and their association with sex, area, salty diet, and exercise.

**Figure 3:**
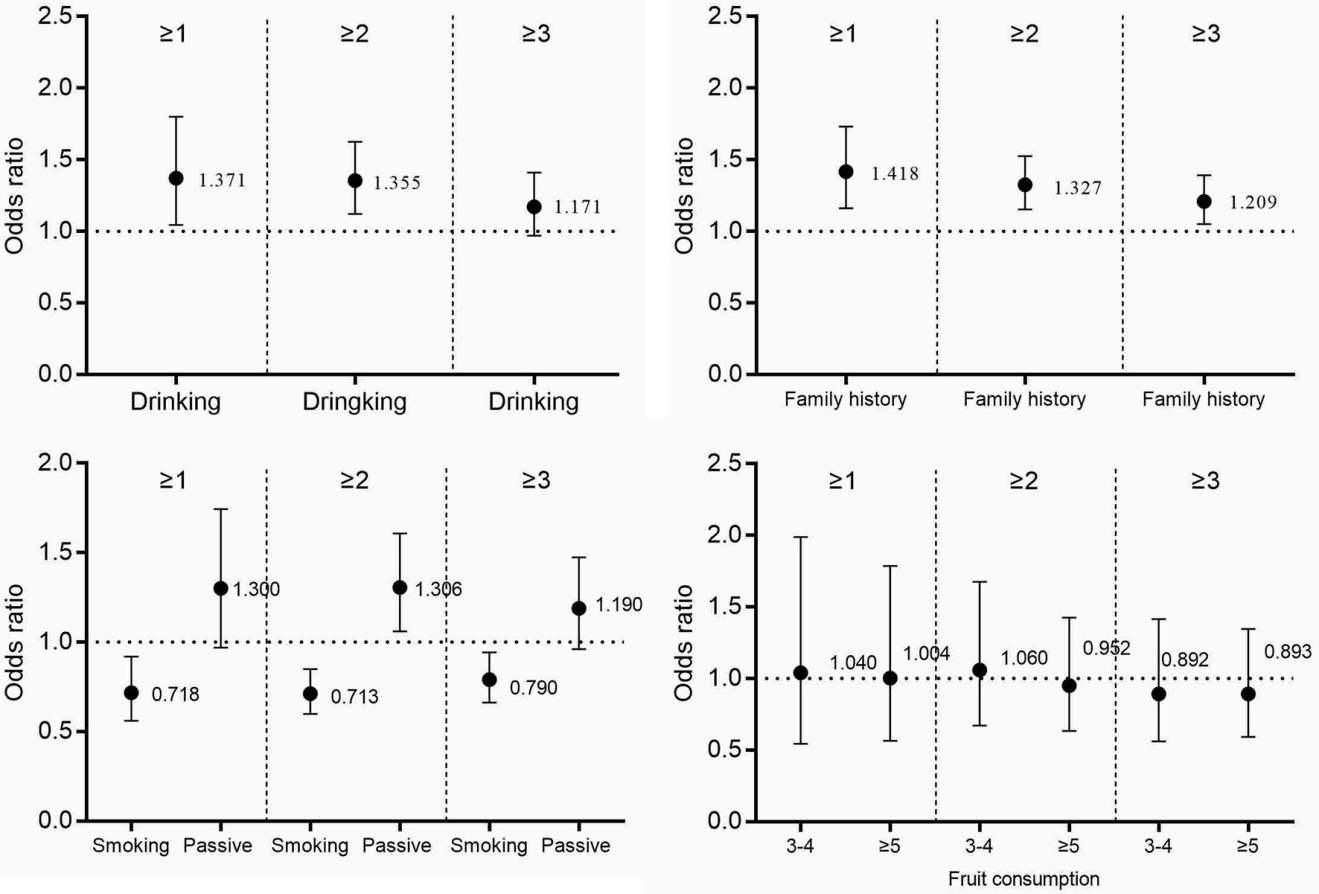
The accumulation of stroke risk factors and their association with drinking, smoking, fruit consumption, and family history of cerebrovascular diseases.

**Figure 4:**
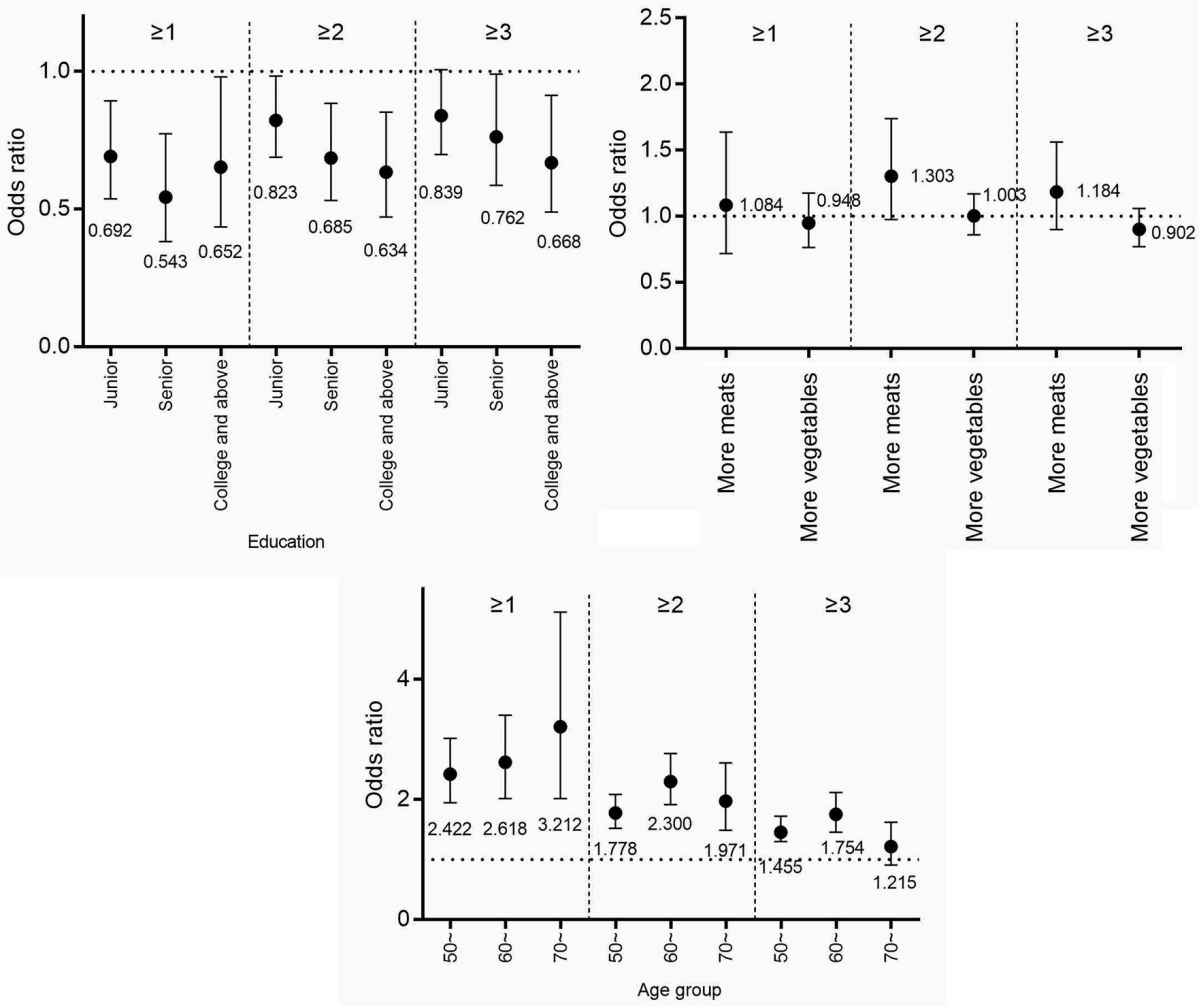
The accumulation of stroke risk factors and their association with age, education, and diet patterns.

Figures 2–4 and Table 3 describes the accumulating of key stroke risk factors and the association with the participants' characteristics. The odds ratios (OR) and 95% confidence intervals (CIs) of having ≥1, ≥2, and ≥3 key stroke risk factors were 1.627 (1.258, 2.103), 1.446 (1.209, 1.728), and 1.394 (1.164, 1.670), respectively, for males compared to females. Similarly, the ORs and 95% CIs of having ≥1, ≥2, and ≥3 key stroke risk factors were 1.227 (1.009, 1.492), 1.256 (1.096, 1.442), and 1.450 (1.262, 1.667), respectively, for partially salty diets compared to normal diets. Compared to people who did not exercise regularly, the ORs and 95% CIs of ≥1, ≥2, and ≥3 key stroke risk factors were 0.693 (0.544, 0.883), 0.800 (0.679, 0.944) and 0.775 (0.659, 0.913), respectively, for those who exercised regularly. When family history of cerebrovascular diseases was the risk factor, and the ORs and 95% CIs were 1.418 (1.162, 1.732), 1.327 (1.154, 1.525), and 1.209 (1.050, 1.393). Data showed that the ORs and 95% CIs of having ≥1, ≥2, and ≥3 key stroke risk factors were 0.718 (0.561, 0.919), 0.713 (0.600, 0.849), and 0.790 (0.662, 0.943), respectively, for smokers compared to non-smokers.

**Table 3:** The accumulation of certain stroke risk factors and the association with demographic characteristics and the subjects' lifestyles.

**Factors**	**OR(95%CI)**		
	≥**1**	≥**2**	≥**3**
**GENDER**			
Female	1	1	1
Male	1.627(1.258,2.103)	1.446(1.209,1.728)	1.394(1.164,1.670)
**AREA**			
Rural	1	1	1
Urban	1.249(0.957,1.631)	1.539(1.270,1.865)	1.314(1.078,1.602)
**AGE (YEAR)**^†^			
40~	1	1	1
50~	2.422(1.944,3.017)	1.778(1.518,2.083)	1.455(1.299,1.723)
60~	2.618(2.014,3.403)	2.300(1.913,2.766)	1.754(1.455,2.114)
70~	3.212(2.016,5.117)	1.971(1.488,2.609)	1.215(0.912,1.619)
**EDUCATION**^‡^			
Primary school and below	1	1	1
Junior middle school	0.692(0.537,0.893)	0.823(0.689,0.983)	0.839(0.699,1.007)
Senior middle school	0.543(0.382,0.773)	0.685(0.531,0.884)	0.762(0.586,0.990)
College and above	0.652(0.435,0.980)	0.634(0.472,0.852)	0.668(0.489,0.913)
**SMOKING**			
No	1	1	1
Yes	0.718(0.561,0.919)	0.713(0.600,0.849)	0.790(0.662,0.943)
Passive	1.300(0.969,1.744)	1.306(1.060,1.608)	1.190(0.961,1.474)
**DRINKING**			
No	1	1	1
Yes	1.371(1.044,1.801)	1.355(1.122,1.636)	1.171(0.970,1.412)
**PARTIALLY SALTY DIET**			
No	1	1	1
Yes	1.227(1.009,1.492)	1.256(1.096,1.442)	1.450(1.262,1.667)
**REGULAR EXERCISE**			
No	1	1	1
Yes	0.693(0.544,0.883)	0.800(0.679,0.944)	0.775(0.659,0.913)
**FAMILY HISTORY OF CEREBROVASCULAR DISEASES**			
No	1	1	1
Yes	1.418(1.162,1.732)	1.327(1.154,1.525)	1.209(1.050,1.393)
**DIETARY PATTERN**			
Balanced	1	1	1
More meats	1.084(0.718,1.637)	1.303(0.977,1.738)	1.184(0.898,1.561)
More vegetables	0.948(0.764,1.176)	1.003(0.860,1.170)	0.902(0.769,1.059)
**FRUIT CONSUMPTION (TIMES PER WEEK)**			
≤ 2	1	1	1
3–4	1.040(0.544,1.989)	1.060(0.671,1.675)	0.892(0.562,1.415)
≥5	1.004(0.565,1.786)	0.952(0.635,1.426)	0.893(0.592,1.346)

† *Trend test. ≥1(p < 0.001);≥2(p < 0.001);≥3(p < 0.001)*.

‡ *Trend test. ≥1(p = 0.014);≥2(p = 0.001);≥3(p = 0.008)*.

## Discussion

This population-based cross-sectional study was the first investigation of stroke and its risk factors in Dehui City in Jilin province, China. As the prevalence and incidence of stroke were significantly increased after 39 years of age, the included participants were over 39 (14). Compared to another population-based cross-sectional survey conducted in Jilin province among residents who were 18–79 years old in 2012, the prevalence of hypertension, hyperlipidemia, and being overweight or obese in this study were higher (57.6 vs. 36.9%; 63.4 vs. 56.81%; 53.3 vs. 46.9%, respectively), while the prevalence of diabetes was lower (9.8 vs. 10.1%) (4, 15). Due to the different age of the two survey, we could not draw a conclusion that the prevalence of hypertension, hyperlipidemia, and being overweight or obese in Dehui City were higher than other places in Jilin province, but we could conclude that the prevalence of diabetes in Dehui City was lower than other areas in Jilin province, as the prevalence of diabetes among people younger than 39 years old was lower than that of people over 39 (16).

The results showed that the prevalence of hypertension, hyperlipidemia, and diabetes had a tendency to increase with age. Studies indicated that advanced age was a risk factor for hypertension, hyperlipidemia, and diabetes, consistent with our findings (16–18). High blood pressure in elderly people could be explained by increased arterial stiffness, which typically accompanies aging and might be exacerbated by high blood pressure (19). Diabetes in the elderly was related to alterations in body composition and to reduced physical activity (20). In this study, the prevalence of overweight or obesity increased first and then decreased with age, and the highest was at 50 to 59 years of age. Ahmed's study reported similar results: 46- to 55-year-olds had the highest prevalence of overweight or obesity (21). It indicates that more attention should be paid to the problem of obesity or overweight in middle-aged people.

A total of 84.1% of the participants in this study had one or more of the four risk factors for stroke which implied that most people over 40 years of age in Dehui City were exposed to the risk of stroke. The risk of accumulating stroke risk factors for males was higher than females. The possible explanation was that the prevalence of overweight or obesity and hypertension were higher in male than in female in this district, but there was no difference in the prevalence of dyslipidemia and diabetes between different sexes (15, 22–24). Our study also indicated that a partially salty diet and lack of physical activity were risk factors for the accumulating of key stroke risk factors. Some scientific advisories recommended that the first line of treatment for hypertension should be lifestyle changes, including low sodium diet and enough physical exercise (25). A low sodium intake was associated with a reduced risk of cardiovascular disease and cerebrovascular disease in adults (26). Therefore, regular exercise and a low salt diet could reduce the risk of the accumulating of key stroke risk factors, thereby reducing the prevalence of stroke in Dehui City. Data showed that smoking was a protective factor for the accumulating of key stroke risk factors in our study. Although smoking was a risk factor for diabetes and hyperlipidemia (27, 28), Mehboudi's study indicated an inverse association between smoking and hypertension in an elderly population in Iran (29), and Wang's study indicated that smokers were less likely to be overweight or obese (15). These findings might explain why smoking was a protective factor for the accumulating of key stroke risk factors in our study. But smoking was an important risk factor for many kinds of diseases, so smoking was still a great threat to the health of residents.

## Limitations

Our study has some potential limitations. Part of the results were based on self-reported data and the recall bias could not be avoided. In addition, the participants were recruited from Dehui City of Jilin province, China, and the conclusion cannot represent the other places in Jilin province. Finally, those who were ill or too weak to complete the interview were not included in our survey.

## Future directions

Special attention should be paid to male or patients with family history of cerebrovascular disease in the primary prevention of stroke. Regular examination of blood pressure, blood fat, blood sugar, and BMI and effective control could reduce their risk of stroke. Health education should be carried out to encourage residents to take salt less diet and regular physical exercise in Dehui City. Although smoking is a protective factor for the accumulation of key stroke risk factors, it is also an important risk factor for many kinds of diseases. So smoking cessation is still crucial to the health of residents.

In conclusion, there was a high prevalence of certain key stroke risk factors among older adults in our study. Male, partially salty diets, and family history of cerebrovascular diseases were risk factors for the accumulating of certain stroke risk factors while regular physical exercise was a protective factor.

## Author contributions

YY and Z-NG handled the conception and design. F-LZ, H-JS, and XS acquired the data. PZ performed the analysis. PZ and HJ drafted the manuscript. Z-NG and YY performed a critical revision.

### Conflict of interest statement

The authors declare that the research was conducted in the absence of any commercial or financial relationships that could be construed as a potential conflict of interest.
