# Supplementary material for: The Accumulation of Key Stroke Risk Factors and Its Association With the Characteristics of Subjects: A Population Based Cross Sectional Study
Source: Front Neurol. 2018 Nov 12;9:949. doi: 10.3389/fneur.2018.00949 (PMC6240764; doi:10.3389/fneur.2018.00949)
Supplement: Supplementary file 1 [file Data_Sheet_1.PDF]

Supplementary Table I. Characteristics of the participants

Characteristics	n	%
**Sex**		
Male	1619	40.0
Female	2433	60.0
**Area**		
Urban	2046	51.0
Rural	1985	49.0
**Age (year)**		
40~	1376	34.0
50~	1372	33.9
60~	1009	24.9
70~	295	7.2
**Education**		
Primary school and below	1446	35.6
Junior middle school	1696	41.9
Senior middle school	537	13.3
College and above	373	9.2
**Smoking**		
Yes	1373	33.9
No	2025	50.0
Passive	654	16.1

**Drinking**		
Yes	1074	26.5
No	2978	73.5
**Partially salty diet**		
Yes	1567	38.7
No	2485	61.3
**Regular exercise**		
Yes	3150	77.7
No	902	22.3
**Family history of cerebrovascular diseases**		
Yes	1397	34.5
No	2655	65.5
**Dietary pattern**		
Balanced	2359	58.2
More meats	264	6.5
More vegetables	1429	35.3
**Fruit consumption (times per week)**		
≤2	113	2.8
3-4	359	8.9
≥5	3580	88.3
